# Antiviral activities of *Artemisia vulgaris L.* extract against herpes simplex virus

**DOI:** 10.1186/s13020-023-00711-1

**Published:** 2023-02-28

**Authors:** Ji Xiao, Ping Liu, Yuze Hu, Tao Liu, Yuying Guo, Pinghua Sun, Junxia Zheng, Zhe Ren, Yifei Wang

**Affiliations:** 1 Jinan Biomedicine Research and Development Center, Department of Cell Biology, College of Life Science and Technology, Jinan University, Guangzhou, 510006 Guangdong People’s Republic of China; 2 Guangdong Province Key Laboratory of Bioengineering Medicine, Jinan University, Guangzhou, China; 3 Guangdong Provincial Biotechnology Drug & Engineering Technology Research Center, Jinan University, Guangzhou, China; 4 National Engineering Research Center of Genetic Medicine, Jinan University, Guangzhou, China; 5 College of Pharmacy, Jinan University, Guangzhou, China; 6 School of Biomedical and Pharmaceutical Sciences, Guangdong University of Technology, Guangzhou, China

**Keywords:** *Artemisia vulgaris L.*, Herpes simplex virus type 1, Extracts, Fr.8.3, Antiviral activity, Molecular docking

## Abstract

**Background:**

*Artemisia vulgaris* L. is often used as a traditional Chinese medicine with the same origin of medicine and food. Its active ingredient in leaves have multiple biological functions such as anti-inflammatory, antibacterial and insecticidal, anti-tumor, antioxidant and immune regulation, etc. It is confirmed that folium *Artemisiae argyi* has obvious anti-HBV activity, however, its antiviral activity and mechanism against herpesvirus or other viruses are not clear. Hence, we aimed to screen the crude extracts (Fr.8.3) isolated and extracted from folium *A. argyi* to explore the anti-herpesvirus activity and mechanism.

**Methods:**

The antiherpes virus activity of Fr.8.3 was mainly characterized by cytopathic effects, real-time PCR detection of viral gene replication and expression levels, western blotting, viral titer determination and plaque reduction experiments. The main components of Fr.8.3 were identified by using LC–MS, and selected protein targets of these components were investigated through molecular docking.

**Results:**

We collected and isolated a variety of *A. vulgaris L.* samples from Tangyin County, Henan Province and then screened the *A. vulgaris L.* leaf extracts for anti-HSV-1 activity. The results of the plaque reduction test showed that the crude extract of *A. vulgaris L.*-Fr.8.3 had anti-HSV-1 activity, and we further verified the anti-HSV-1 activity of Fr.8.3 at the DNA, RNA and protein levels. Moreover, we found that Fr.8.3 also had a broad spectrum of antiviral activity. Finally, we explored its anti-HSV-1 mechanism, and the results showed that Fr.8.3 exerted an anti-HSV-1 effect by acting directly on the virus itself. Then, the extracts were screened on HSV-1 surface glycoproteins and host cell surface receptors for potential binding ability by molecular docking, which further verified the phenotypic results. LC–MS analysis showed that 1 and 2 were the two main components of the extracts. Docking analysis suggested that compounds from extract 1 might similarly cover the binding domain between the virus and the host cells, thus interfering with virus adhesion to cell receptors, which provides new ideas and insights for clinical drug development for herpes simplex virus type 1.

**Conclusion:**

We found that Fr.8.3 has anti-herpesvirus and anti-rotavirus effects. The main 12 components in Fr.8.3 were analyzed by LC–MS, and the protein targets were finally predicted through molecular docking, which showed that alkaloids may play a major role in antiviral activity.

## Introduction

### Overview of HSV-1

Herpes simplex virus (HSV) is a common human pathogenic virus belonging to the herpesvirus subfamily. Herpes simplex viruses (HSV-1 and HSV-2) are linear double-stranded DNA viruses consisting of a four-layer structure and at least 18 proteins with a complex spherical structure [1, 2]. Physical and chemical analysis showed that the length of the HSV DNA is approximately 150 KB, while the actual sequencing results have shown that the length of the HSV-1 DNA is 152260 bp (17 strains, GenBank ID: X14112) [3]. HSV is mainly divided into two serotypes, type I (HSV-1) and type II (HSV-2). The viral genome structure and DNA sequence of these two serotypes have a very high homology and complex structures [4]. The proliferation process of HSV-1 includes adsorption, membrane penetration, nuclear transport, DNA replication, gene transcription, protein synthesis, nucleocapsid assembly and virus release [5].

### Research status of HSV-1

According to published reports, the infection rate of herpes simplex virus type I in adults is 60–95%, and the herpes simplex virus type II infection rate is 60% [6]. HSV-1 can cause herpes simplex virus keratitis, herpes simplex conjunctivitis, cold sores and other diseases. Recurrent eye infections caused by HSV-1 are one of the main causes of blindness [7]. HSV-1 is a common pathogen causing central nervous system infections, which can lead to severe focal necrotizing encephalitis with high clinical mortality and poor prognosis, and patients are often left with many neurological defects [8]. HSV-1 can establish latent infection in trigeminal ganglion cells to escape immune system attack. HSV-1 is also the main pathogen that leads to concurrent infection with other viruses, such as human immunodeficiency virus (HIV), and affects HIV infection, morbidity and prognosis [9]. Due to the neurotropic nature of herpesviruses, studies have shown that HSV-1 can also cause fatal encephalitis and lead to a high mortality from encephalitis [10]. According to the latest report of the World Health Organization, two-thirds of people under the age of 50 in the world are infected with HSV-1, the incidence of neurogenic encephalitis caused by HSV-1 infection is increasing year by year, and the fatality rate can reach 70% in the absence of effective treatment [1, 5]. Currently, nucleosides and their analogs, such as acyclovir (ACV) and valaciclovir (VCV), are commonly used in clinical treatment [11]. ACV targets viral DNA polymerase and thymic nucleoside kinase. It works by blocking viral DNA replication [12]. The newly developed nucleoside analogs famiclovir (FCV) and valaciclovir are more bioavailable than acyclovir [13, 14]. However, these drugs neither completely eliminate the virus from the host nor prevent the recurrence of HSV. They are not effective in the early stages of infection. With the widespread use of antiviral drugs, this has led to mutations in the virus, leading to the increasing resistance of HSV-1 [12, 15–17]. Therefore, it is increasingly urgent to explore new drugs, such as other nonnucleoside analogs and natural compounds, that are effective against HSV-1.

### Components and biological functions of *Artemisia vulgaris L.*

*Artemisia argyi *Levl. et Vant. belongs to the Compositae family, genus Artemisia, which contains more than 500 varieties, including *Artemisia vulgaris L.,* perennial herbs and semishrubby plants that have a strong aroma. Its taproots are distinct and slightly long, with a diameter of 1.5 cm and lateral roots. It is widely distributed in Mongolia, Korea, Russia (Far East) and China and found throughout most of China except for extremely arid and alpine areas [18, 19]. Since ancient times, *Artemisia argyi *Levl. et Vant., as a traditional Chinese medicine that is used as medicine and food, has the effects of warming the meridian, relieving pain, dispelling dampness and dispersing cold [20]. *A. vulgaris L.* leaf is the dry leaf of the herbaceous plant *Artemisia vulgaris L.*, and its main components include terpenoids, flavonoids, phenolic acids and polysaccharides [21–23]. In addition, it also contains phenylpropanoids, steroids and fatty acids [24]. The active components of *Foliu*m *A. argyi* have different biological functions and pharmacological effects. Studies in vitro and in vivo have shown that the phytochemicals of *A. argyi* have a variety of health-promoting potential, including antioxidant, anticancer, anti-inflammatory, immunomodulatory, neuroprotective, anticoagulant, anti-osteoporosis, and antibacterial and insecticidal activities [20]. The essential oil from *A. argyi* (AAEO) has anti-inflammatory and blood-activating effects [25]. Studies have shown that the essential oil from *Folium A. argyi* has antibacterial activity, and its mechanism may involve damaging the cell membranes, resulting in leakage of proteins, K + and other intracellular substances [26]. In addition, *Folium Artemisiae argyi* essential oil also has strong antioxidant activity and can be used as an inhibitor of melanin production and a natural antioxidant for skin care products [27]. Polysaccharide from *Folium A. argyi* (AAFP) has antitumor and immunomodulatory activities [28]. In addition, some studies have reported its antioxidant and cellular anti-inflammatory activities [29]. Therefore, AAFP has potential application value as an antioxidant and immune enhancer and has great development potential as a functional food and medical product. The flavone of *Folium A. argyi* (FEAA) has anticoagulant [30], antimutagenesis [31], antioxidant [29, 32] and antitumor effects [33], and numerous studies have shown that it has important medicinal value. Studies have shown that AgNPs, a kind of metal nanoparticle, synthesized with *Folium A. argyi* extract showed a high antimicrobial activity against common gynecological pathogens, such as *Acinetobacter baumannii*, *Staphylococcus aureus*, *Escherichia coli* and *Candida albicans* [34]. Finally, Zhao Zhihong et al. have shown that *Folium A. argyi* has obvious anti-hepatitis B virus (HBV) activity by demonstrating that the ethyl acetate extract of *Folium A. argyi* had obvious anti-HBV activity in vitro and showed a strong inhibition of HBV surface antigen and HBV E antigen, as well as inhibition of HBV DNA [35]. However, there are no reports that *A. argyi* can inhibit herpesviruses.

## Materials and methods

### Plant material

The *Artemisia vulgaris L.* used in this experiment was collected from Tangyin, Henan Province. *A. vulgaris L.* belongs to the Artemisia genus of the Compositae family. It is a perennial herb or slightly semishrubby plant with a strong fragrance. The taproot of the plant is obvious and slightly long, with many lateral roots, and the plant is 1.5 cm in diameter. This plant often has recumbent rhizomes and vegetative shoots. The stem of the plant grows singly or clustered, with distinct longitudinal ribs; their color is brown or tawny brown, the stem is slightly woody at the base, grassy above, with a few short branches, and the length is 3–5 cm. The stems and branches have gray arachnid-like hair. The leaves are thick, covered with gray–white pubescence above, with white glandular spots and small concave spots, and densely covered with gray–white spider silky hairs on the abaxial surface; the basal leaves have long stalks, fading at the anthesis. The shape of the lower stem leaves is suborbicular or broadly ovate, pinnate and deeply lobed, with 2–3 lobes on each side; the lobes’ shapes are oval or obovate and long elliptic, with 2–3 small teeth on each of them. The main and lateral veins on the back of the stem are dark brown or rusty, and the length of petiole is 0.5–0.8 cm. Middle leaves are ovate, triangular-ovate or subrhomboid, with one (to two) pinnate deeply lobed to hemiclobed, with 2 or 3 lobes on each side; the shape of lobes is ovate, ovate-lanceolate or lanceolate, the leaf base is broadly cuneate tapering into a short petiole, and the leaf veins are conspicuous, bulging on the back, with the leaf stalk base usually without false stipes or very small false stipes. Upper leaves and bracts are pinnatifid lobed, deeply 3-lobed, 3-lobed, or not divided, but the shape is elliptic, long elliptic lanceolate, lanceolate or linear-lanceolate.

The shape of the capitulum is oval, sessile or subsessile, with several to more than 10 of them arranged in small spikes or compound spikes on branches, and usually reconstituted in narrow pinnacle-shaped panicles on the stems, with capitulum decumbent after flowers. Involucral bracts have 3–4 layers, arranged in a imbricate shape, the outer involucral bracts are small, in ovate or narrowly ovate shapes, abaxially densely covered with grayish white silky spider hair, and the edge is membranous; the middle involucral bracts are longer than the outer ones, with a long ovate shape, abaxially covered with silky spider silky hair, and the inner involucral bracts are thin, abaxially nearly glabrous. The inflorescence torr is small, (6–10 in female flowers). The purple corolla is narrowly tubular, 2-lobed on the leaf, and the style is slender, 2-forked at the apex. The inflorescence of bisexual flowers is 8–12, the corolla is tubular or goblet shaped, with glandular spots on outside, and the leaves are purple. The anthers are narrowly linear, with long triangular and pointed apical appendages and inconspicuous pinnacles on the base of anther. The achene has a long ovate or oblong shape. The flowering and fruiting period is from July to October.

### Artemisia vulgaris L. extraction

*A. vulgaris L.* was collected from Tangyin, Henan Province. First, *A. vulgaris L. leaves* were dried and powdered to approximately 40 eyes. Then, 2 kg of *A. vulgaris L.* leaf powder was loaded into the extraction kettle, and 16 L of 95% ethanol was added at a ratio of 1:8 to fully infiltrate. The extraction solution was collected, and the solvent was concentrated for recovery. The crude ethanol extract of the *A. vulgaris L.* leaves were extracted 3 times. The crude ethanol extract of *A. vulgaris L.* leaves were suspended in twice the weight of pure water, and petroleum ether of equal volume (60–90 °C boiling range) was added. After fully stirring for 5 min, the mixture was left standing until obvious stratification appeared, and the petroleum ether extract of the upper layer was collected. Petroleum ether extraction was repeated 3 times before the isopyknic ethyl acetate extraction; after fully mixing, the mixture was allowed to stand for 5 min, until obvious stratification occurred, after which the upper ethyl acetate extract was collected. After repeated extraction 3 times, the ethyl acetate extract was concentrated to obtain the ethyl acetate extract of the *A. vulgaris L.* leaves. Finally, the petroleum ether layer, ethyl acetate layer and water layer were obtained. Then, the ethyl acetate extract of the *A. vulgaris L.* leaves were dissolved in methanol, and 100 mesh silica gel at 1.2 times the weight was added for sample mixing. A silica gel chromatography column with 20 times the sample volume was used, and dichloromethane was used for activation and balancing. The dried samples were dried on a silica column bed, and dichloromethane: methanol (100:0, 99.5:0.5, 99:1, 74:1, 64:1, 49:1, 33:1, 97:3, 95:5, 90:10, 80:20, 50:50) was used. The Fr. 1-Fr. 9 components were obtained by gradient elution and combined by thin layer silica gel chromatography. Fr. 8 (21.6 g) was mixed with 1.2 times the volume of MCI, and a sample volume of 15 times the MCI column was loaded. The elution gradient was methanol: water (10%-100%), and the Fr.8.1-Fr.8.7 components were obtained.

### LC–MS Analysis of alkaloids

The extract was diluted 5 times with MeOH. An aliquot of 5 μL was analyzed by using an UltiMate 3000 UPLC system (Thermo, MA, USA) with an ACQUITY UPLC HSS T3 (1.8 μm 2.1 × 100 mm) column coupled to a QTOF 5600 system (AB SCIEX, CA, USA). The flow rate was set at 400 µL/min using H_2_O and acetonitrile as solvent A and solvent B, respectively. When using ESI positivity, the solvent was supplemented with 0.1% formic acid, but when using ESI negativity, only solvent A was supplemented with 2 mM ammonium acetate. The adopted gradient was set as follows: 5% B was maintained for approximately 1.5 min, followed by an increase from 5 to 10% B in 1 min, 10% to 40% B in 11.5 min, and 40% to 95% B in 8 min, which was maintained for approximately 3 min, after which there was a decrease from 95 to 5% B in 1 min, and then an isocratic flow at 5% B for 4 min. The mass spectrometer was operated in full scan by TOF–MS acquisition and product ion scans by means of information-dependent acquisition (IDA). TOF–MS acquisition was set under the following conditions: positive-ion mode, ion source gas 1 (nitrogen gas, 60 psi), ion source gas 2 (nitrogen gas, 60 psi), curtain gas 1 (nitrogen gas, 35 psi), temperature at 650 °C, ion spray voltage floating (ESI + : + 5000 V; ESI−: − 4000 V), and collision energy (CE, 30 eV). The IDA mode of data acquisition consisted of a full MS1 scan and information-dependent trigger MS/MS fragmentation events. The mass was scanned in a range from 50 to 1200, a threshold above 100 cps of ions was selected for the information-dependent trigger MS/MS fragmentation events, and the speed of the 10 ms/ms scan required 50 ms. The data was analyzed by MSDIAL, the accurate mass tolerance was set as MS1: 0.01 Da and MS2: 0.05 Da, and the identification score cut-off was set as 60%.

### Molecular docking

#### Ligand preparation

The geometrically optimized structures were retrieved as MOL2 types and further analyzed using the LigPrep application implemented in the Schrodinger suite of programs. The ionization state was specified at pH = 7.00 ± 2.0 by applying Epik based on the Hammett and Taft methodologies. Desalination was carried out by the program default. All 50 possible conformations were produced for each compound at pH = 7.00 in the OPLS-2005 force field. The obtained ligands were further prepared for application in the docking calculations.

#### Generation of the grid

The key step in predicting ligand–receptor binding is to fully generate the grid. Herein, a 3D boundary for ligand binding was produced by Glide (Maestero version 12.5, Schrodinger). First, using the Protein Preparation Wizard, the following steps were performed. (1) The original hydrogen was removed, and only the polar hydrogens were subsequently added. (2) Atomic charges and bond orders were assigned. (3) All water molecules that were not involved in ligand binding or had less than 5 Å H-bonds with nonwater were removed. (4) The N- and C-termini were capped. (5) Disulfide bonds were generated between sulfur atoms (within 3.2 Å). (6) Epik was applied to generate possible protonation states at a neutral pH. (7) H-bonds were assigned and further optimized by PROPKA at pH = 7.0. Then, the structure was minimized with the OPLS-2005 force field. Finally, Glide was used to generate a grid box on the outer domain of the receptors. Since our aim was simply to estimate the dependence of these compounds on the investigated receptors, the position of the grid boxes was chosen manually by PlayMolecule (https://playmolecule.com/).

#### Molecular interaction and docking studies of *Artemisia argyi* compounds with three target proteins

After grid generation, ligand docking was finished according to Glide’s protocol. HSV-1 glycoprotein D (gD) bound to the human receptor nectin-1 (pdb ID: 3 sku) was selected as a model for docking of derived Artemisia argyi compounds to investigate the binding domains of gD to its host receptor nectin-1. In addition, the extracellular structure of glycoprotein B (gB) of HSV-1 (pdb ID: 4bom) was used to assess the attachment of the studied natural products to gB. Finally, the crystal structure of nectin-1 (PDB code: 4fmf) was used as the representative host receptor. It was found that the docking score is a spotlight parameter to evaluate the conformations to generate the best conformations for each ligand as an output. Here, the output of standard precision (SP) docking was presented as extra precision (XP) docking.

### Cell lines and virus

African green monkey kidney cells (Vero; ATCC CCL81) were cultured in Dulbecco’s modified eagle medium (DMEM) supplemented with 10% FBS in a constant temperature cell incubator with 5% CO_2_ at 37 °C. MA-104 cells were cultured in Minimum Essential Medium (MEM) supplemented with 10% FBS in a constant temperature cell incubator with 5% CO_2_ at 37 °C. The maintenance medium used for virus dilutions was DMEM supplemented with 2% FBS. HSV-1/F (ATCC VR-733), acyclovir-resistant strains HSV-1/Blue and HSV-1/153, TK mutants derived from HSV-1 (KOS), HSV-2 and rotavirus (RV) were all preserved in our lab.

### Cytotoxicity test and cytopathic effect assessment

The cytotoxicity of the extracts was assessed by the Cell Counting Kit-8 (Beyotime, China, C0037) assay. Antiviral activity was evaluated by the cytopathic effect (CPE) assessment [36]. Vero cells were seeded into 96-well plates at a density of 1 × 10^4^ cells/well. Cells were grown in 96-well plates to a single layer, then the growth medium was removed and replaced with samples of maintenance medium containing an 11-concentration series of extract dilutions (starting with the highest concentration that was nontoxic); each well contained 50 μL maintenance medium, to which 50 μL of virus dilution (MOI = 0.1) was then added. Each group had eight replicate wells, and a virus control group and a normal control group were also set up; all wells were incubated for 48 h. The cytopathic changes were observed under an inverted microscope. The CPE of each well was observed and recorded when the cytopathic rate of the virus control group was more than 75% and that of the normal control group was normal. CPE was recorded as follows: no cytopathic lesions, “−”; 1–25% cytopathic lesions, “ + ”; 26%-50% cytopathic lesions, “ +  + ”; 51–75% cytopathic lesions, “ +  +  + ”; 76–100% cytopathic lesions, “ +  +  +  +  + ”.

### Plaque reduction assay

Vero cells were seeded into 12-well plates at a density of 2.5 × 10^5^ cells/well. When cells reached 90–100% confluence, the medium was discarded, and cells were infected with HSV-1/F (30–40 PFUs per well) for 2 h. Then, the viral suspension was removed, and the cells were incubated with 1 mL of drystrip cover medium (50% sodium carboxymethylcellulose and 50% 2 × DMEM) containing various concentrations of inhibitors. After 72 h, the drystrip cover medium was gently removed, and all wells were washed three times with phosphate buffered saline (PBS). Cells were fixed with 4% paraformaldehyde for 30 min, dyed with 1% crystal violet for 30 min and then washed with tap water. The plaque numbers were counted, and the percentage of inhibition was calculated.

### RNA/DNA extraction and quantitative real-time PCR

The cells were treated on the basis of the diverse experimental requirements. Total RNA was extracted using TRIzol, and the RNA concentration was measured at 260/280 nm using a Nano Photometer P330 spectrophotometer (IMPLEN, Munich, Germany). One microgram of RNA from each sample was reverse transcribed into cDNA using a reagent kit (PrimeScript RT Reagent Kit, Takara, Shiga, Japan). Afterward, the mRNA expression levels of viral genes were analyzed using a Bio–Rad CFX96 real-time PCR system (Bio–Rad, CA, USA). The expression level of GAPDH was used as a reference. The primer sequences were as follows: HSV-1 UL54 F (5ʹ-TGGCGGACATTAAGGACATTG-3ʹ), UL54 R (5ʹ-TGGCCGTCAACTCGCAGA-3ʹ), UL52 F (5ʹ-GACCGACGGGTGCGTTATT-3ʹ), UL52 R (5ʹ-GAAGGAGTCGCCATTTAGCC-3ʹ), UL27 F (5ʹ-GCCTTCTTCGCCTTTCGC-3ʹ), UL27 R (5ʹ-CGCTCGTGCCCTTCTTCTT-3ʹ), ICP0 F (5ʹ-CCCACTATCAGGTACACCAGCTT-3ʹ), ICP0 R (5ʹ-CTGCGCTGCGACACCTT-3ʹ), GAPDH F (5ʹ-GTCATTGAGAGCAATGCCAG-3’) GAPDH R (5ʹ-GTGTTCCTACCCCCAATGTG-3ʹ).

The viral DNA copy numbers of the viral genes UL54, UL52 and UL27 were assessed. Total DNA was extracted using a TIANamp Virus DNA/RNA Kit (Transgene, Beijing, China), and targeted genes were analyzed by quantitative real-time PCR (Q-PCR). The Q-PCR data analysis followed the procedure described in manufacturer's specification.

### Western blotting

Vero cells were infected with HSV-1/F (MOI = 1) and treated with Fr.8.3 at multiple concentrations (0 μg/mL, 5 μg/mL, 10 μg/mL, 20 μg/mL and 40 μg/mL). After 24 h, the cells were collected and lysed in RIPA lysis buffer (Beyotime, China) containing 2% PMSF and separated by 10% gradient SDS–PAGE. Then, the samples were transferred to PVDF membranes and incubated with primary antibodies overnight at 4 °C, followed by incubation with appropriate secondary antibodies (Beyotime, China) (1:6000–8000 dilutions) for 60 min at room temperature. Target proteins were detected by enhanced chemiluminescence, and GAPDH was used as an internal standard.

### Viral inactivation, attachment and penetration

#### Viral inactivation test

First, after digestion by trypsin, Vero cells were added to a 12-well culture plate at 1 mL/well, with a cell density of 2 × 10^5^ cells/mL. Different concentrations of drug and virus dilutions (100 μL each) were mixed together and incubated at 37 ℃ for 2 h. The virus control group was mixed with maintenance solution and incubated at 37 °C for 2 h. Then, the cell culture solution was discarded from the plate, which was washed once with PBS, and then the incubation solution was added. Three replicate wells were used for each drug concentration, as well as for the positive drug control group, virus control group and normal cell control group. The cultures were incubated for 2 h in a 5% CO_2_ incubator at 37 ℃. The mixture of drug and virus was aspirated and discarded, the plate was washed once with PBS, and 1 mL/well of complete covering medium was added. After 72 h of culture in a 5% CO_2_ incubator at 37 °C, the cells were fixed with 10% formaldehyde for 30 min, dyed with 1% crystal violet for 30 min, washed with tap water and dried. The number of plaques was counted, and the plaque inhibition rate was calculated.

#### Viral attachment test

First, after digestion by trypsin, Vero cells were added to a 12-well culture plate at 1 mL/well, with a cell density of 2 × 10^5^ cells/mL. After the cells grew into monolayers, the culture plate was precooled at 4 °C for 1 h. Then, the plate was washed once with PBS, and 100 μL of drug and virus diluent (30 PFUs/well) was added to each well. Three replicate wells were used for each drug concentration, as well as for the positive drug control group, virus control group and normal cell control group. The plates were incubated at 4 ℃ for 80 min. Then, the mixtures of drugs and viruses were discarded, the plate was washed with PBS pH 7.2 three times, and 1 mL/well of complete covering fluid was added. After 72 h of culture in a 5% CO_2_ incubator at 37 °C, the cells were fixed with 10% formaldehyde for 30 min, dyed with 1% crystal violet for 30 min, washed with tap water and dried. Finally, the number of plaques was counted, and the plaque inhibition rate was calculated.

#### Viral penetration test

First, after digestion by trypsin, Vero cells were added to a 12-well culture plate at 1 mL well, with a cell density of 2 × 10^5^ cells/mL. After the cells grew into monolayers, the culture plate was precooled at 4 °C for 1 h. The plate was washed once with PBS, 100 μL of virus dilution (30 PFUs/well) was added, and the plate was incubated at 4 °C for 2 h. Then, 100 μL of diluted virus was added to each well and incubated at 37 °C for 10 min. Three wells were used for each drug concentration, and a positive drug control group, virus control group and normal cell control group were also used simultaneously. Then, PBS pH 3.0 (200 μL/well) was added to the plate for 1 min, followed by the addition of PBS pH 11.0 (200 μL/well) for 1 min, which was then discarded, after which 1 mL/well complete covering medium was added. After 72 h of culture in a 5% CO_2_ incubator at 37 °C, the cells were fixed with 10% formaldehyde for 30 min, dyed with 1% crystal violet for 30 min, washed with tap water and dried. Finally, the number of plaques was counted, and the plaque inhibition rate was calculated.

### Statistical analysis

Data are presented as the mean ± SD. Data were analyzed by one-way analysis of variance or Student’s t test as appropriate, and the level of significance was set at p < 0.05 (*), p < 0.01 (**), or p < 0.001 (***).

## Results

### Extraction and isolation process of Fr.8.3 and antiviral drug screening

**Fig. 1:**
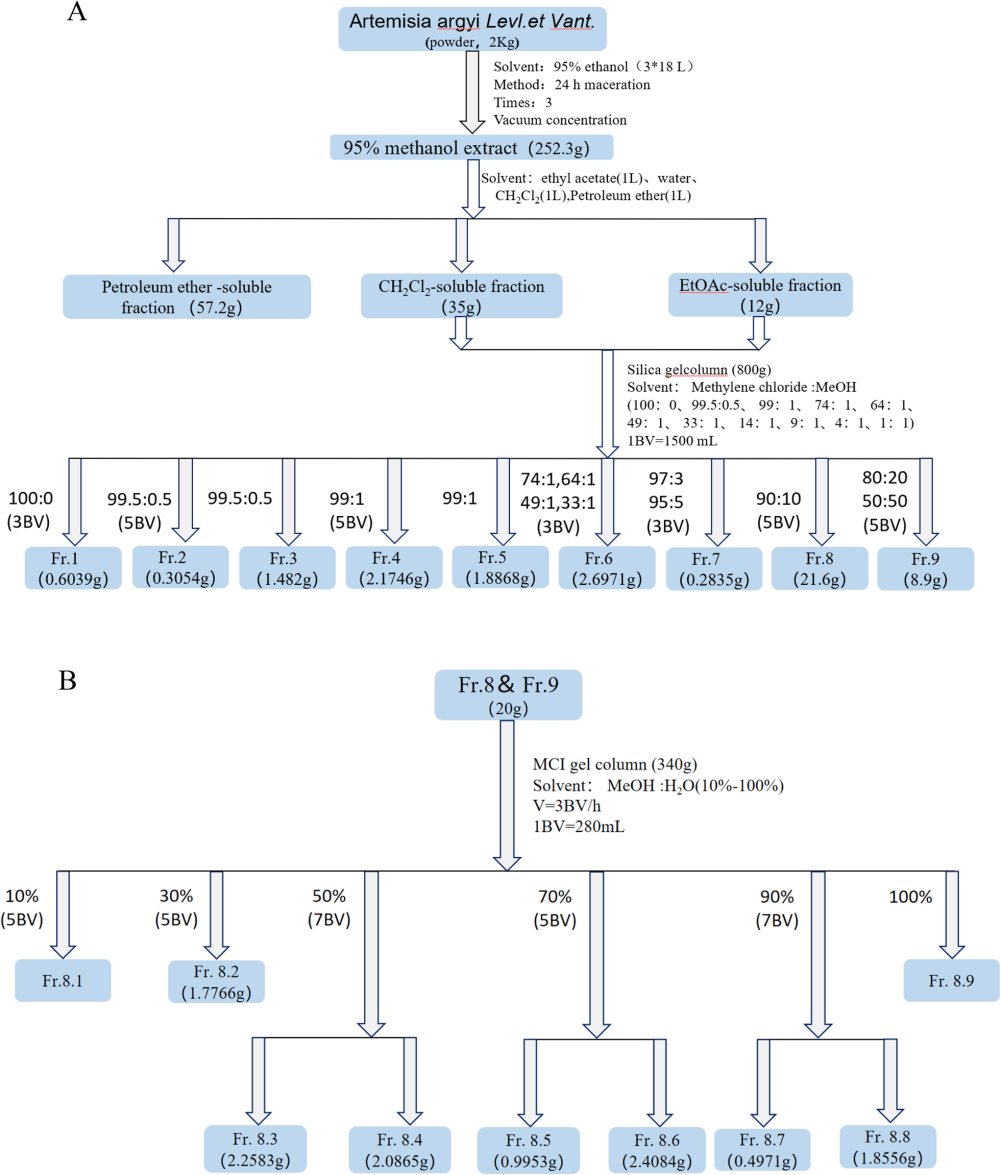
Extraction and isolation process of Fr. 8.3 from *Artemisia vulgaris L.* leaves

We carried out the chemical separation and extraction processes of *Artemisia vulgaris L.* from Tangyin County, Henan Province. The specific method is detailed in the Materials and Methods section, and the separation process is summarized in Fig. 1.

To determine whether the extracts have antiherpesvirus activity, we screened the abovementioned *Artemisia vulgaris L*. leaf extracts for antiviral activity. The cytopathic effect (CPE) results are shown in Table 1, which indicate that Fr.8.3 had good antiviral activity and that the cytopathic rate was 1–25% at a concentration of 40 μg/mL. These results suggest that Fr.8.3 has good anti-HSV-1 activity.

**Table 1:** Antiviral activity screening of extracts from *Artemisia vulgaris L*

Extract Number (in a DMSO solvent)	Extract concentration (μg/mL)	Extract concentration (μg/mL)	Extract concentration (μg/mL)	Extract concentration (μg/mL)	Extract concentration (μg/mL)	Antiviralactivity
Fr.8.1 52.3 mg/mL	40 μg/mL (+ + + +)	20 μg/mL (+ + + +)	10 μg/mL (+ + + +)	5 μg/mL (+ + + +)	2.5 μg/mL (+ + + +)	No
Fr.8.2 23.2 mg/mL	40 μg/mL (+ + + +)	20 μg/mL (+ + + +)	10 μg/mL (+ + + +)	5 μg/mL (+ + + +)	2.5 μg/mL (+ + + +)	No
Fr.8.3 54.8 mg/mL	40 μg/mL ( +)	20 μg/mL (+ + + +)	10 μg/mL (+ + + +)	5 μg/mL (+ + + +)	2.5 μg/mL (+ + + +)	Yes
Fr.8.4 34 mg/mL	40 μg/mL (+ + + +)	20 μg/mL (+ + + +)	10 μg/mL (+ + + +)	5 μg/mL (+ + + +)	2.5 μg/mL (+ + + +)	No
Fr.8.5 85 mg/mL	40 μg/mL (+ + + +)	20 μg/mL (+ + + +)	10 μg/mL (+ + + +)	5 μg/mL (+ + + +)	2.5 μg/mL (+ + + +)	No
Fr.8.6 36 mg/mL	40 μg/mL (+ + + +)	20 μg/mL (+ + + +)	10 μg/mL (+ + + +)	5 μg/mL (+ + + +)	2.5 μg/mL (+ + + +)	No
Fr.8.7 34.8 mg/mL	40 μg/mL (+ + + +)	20 μg/mL (+ + + +)	10 μg/mL (+ + + +)	5 μg/mL (+ + + +)	2.5 μg/mL (+ + + +)	No

### Toxicity effects of Fr.8.3 on different cell lines

**Fig. 2:**
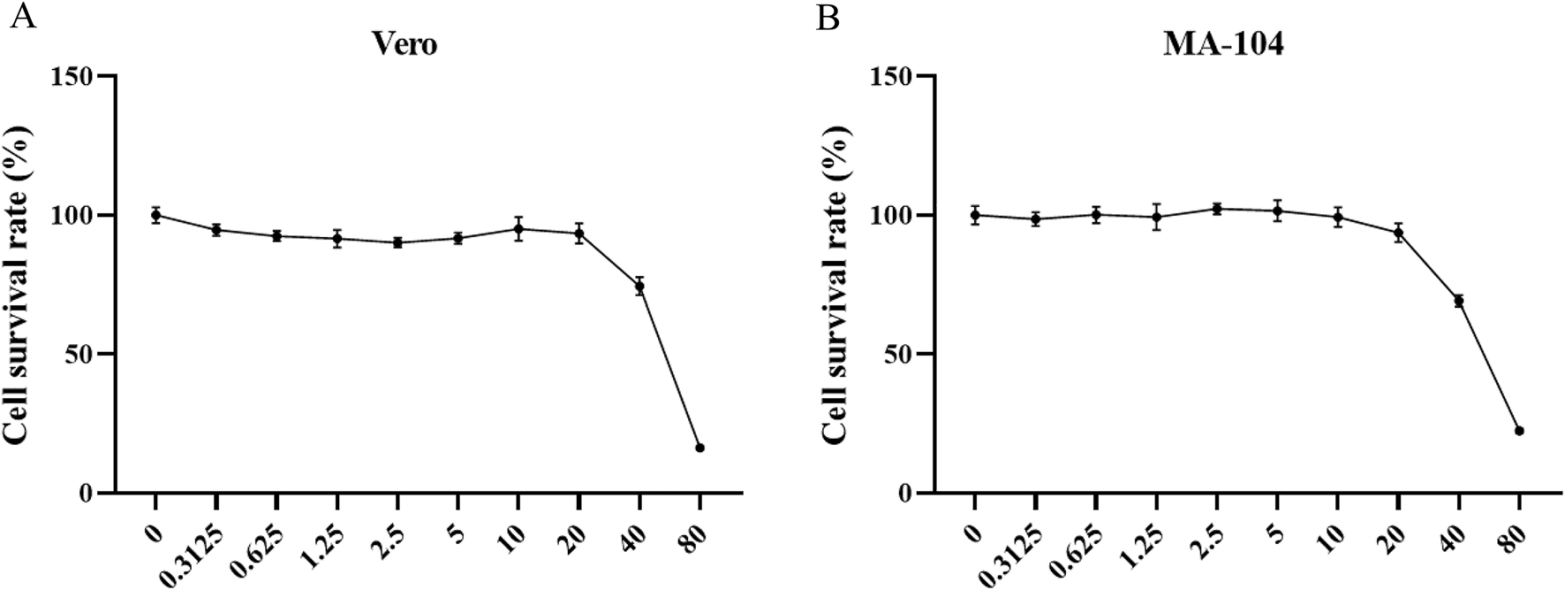
Cytotoxicity of Fr.8.3 on different cell lines. **A** Vero cells (IC_50_ = 85.6 μg/mL), **B** MA-104 cells (IC_50_ = 62.9 μg/mL). Data represent the mean ± SD

To assess the toxicity of Fr.8.3 on different cell lines, a CCK-8 assay was used to determine the toxicity of Fr.8.3 on two cell lines, which was represented by the 50% inhibition rate (IC_50_)/50% cell survival rate (CC_50_). As seen from the cell survival rate of each cell line in Fig. 2, the trend in cytotoxicity of Fr.8.3 on both cell lines was consistent; that is, with the increase in drug concentration, the cell survival rate decreased. The half-inhibition rates (IC_50_) of Fr.8.3 on Vero and MA-104 cells were 85.6 and 62.9 μg/mL, respectively (Fig. 2). These results suggest that Fr.8.3 has a low cytotoxicity, accompanied by a strong antiviral activity.

### Comprehensive anti-HSV-1 effect of Fr.8.3

**Fig. 3:**
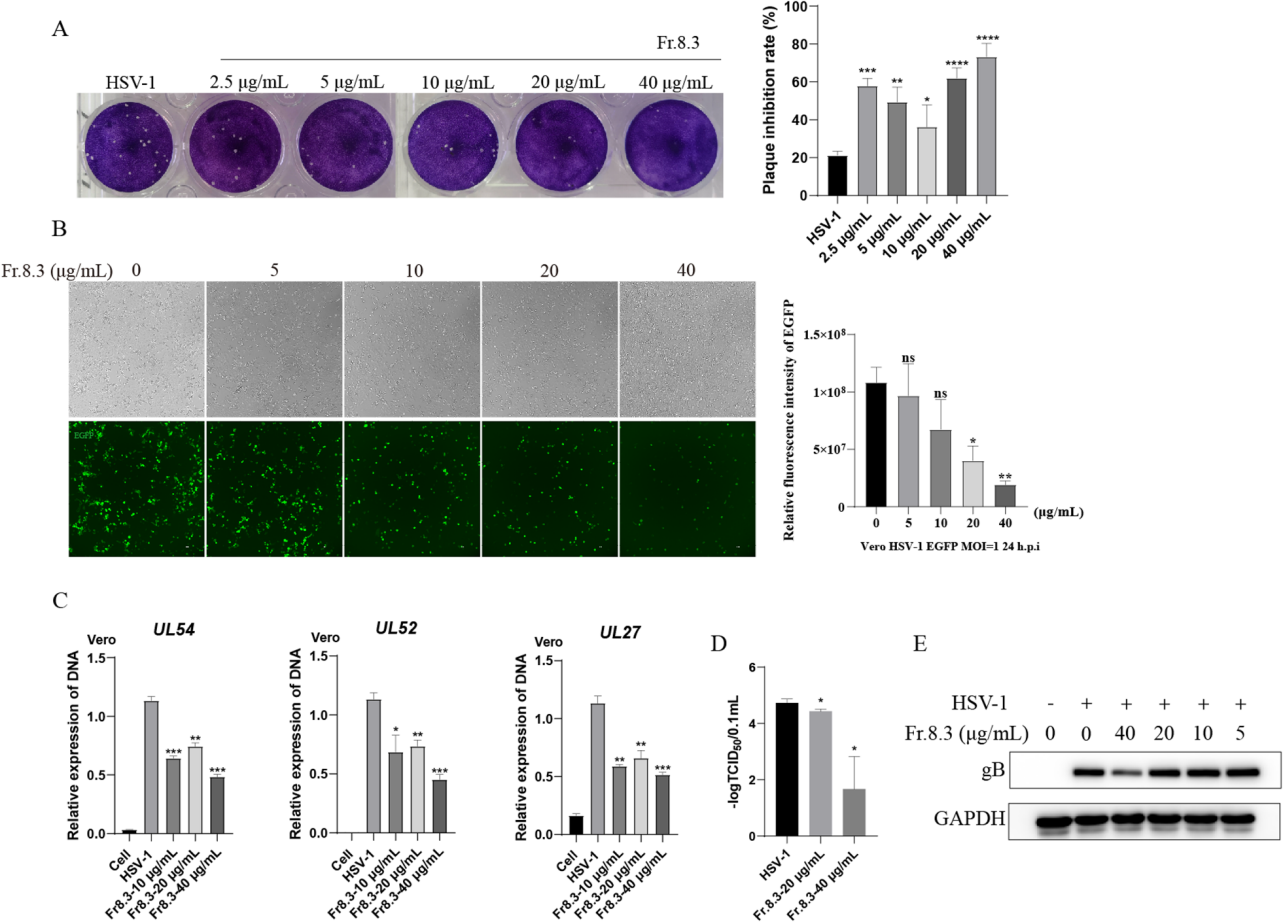
Comprehensive anti-HSV-1 activities of Fr.8.3. **A** plaque reduction test, **B** fluorescence detection experiment. HSV-1 fluorescence reporter strains tagged with EGFP were constructed to assess the signal value of fluorescence intensity, and then the inhibitory effect of Fr.8.3 on HSV-1 was evaluated, **C** real-time fluorescence quantitative amplification reactions (Q-PCR) assessing the DNA level, **D** viral titers of HSV-1, **E** western blotting. The results presented were obtained from three independent experiments. Data represent the mean ± SD. Statistical differences were analyzed via Student’s t test, and a value of p ≤ 0.05 was considered significant (^*^p ≤ 0.05, ^**^p ≤ 0.01, ^***^p ≤ 0.001)

To further evaluate the anti-HSV-1 activity of Fr.8.3, we measured the anti-HSV-1 activity of Fr.8.3 on Vero cells by the following methods. According to the results of the plaque reduction test, the concentration of Fr.8.3 at 40 μg/mL had an obvious anti-HSV-1 effect, the inhibition rate of plaque was more than 70% (Fig. 3A). In addition, we further verified the above conclusion by constructing HSV-1 fluorescence reporter strains labeled with EGFP. The results showed that the fluorescence intensity of the EGFP fluorescence reporter strains was significantly reduced when the concentration of Fr.8.3 was 40 μg/mL, and the results of significance difference analysis were consistent with this (Fig. 3B). Fr.8.3 at 40 μg/mL significantly inhibited DNA replication of the HSV-1 genomic DNA expression levels and decreased HSV-1 titer to a certain extent (Fig. 3C, D). Finally, western blot results also showed that the concentration of Fr.8.3 at 40 μg/mL significantly inhibited the expression level of HSV-1 proteins (Fig. 3E).

In conclusion, Fr.8.3 can inhibit HSV-1 at the plaque production and DNA, RNA and protein levels, indicating that it has a comprehensive anti-HSV-1 effect.

### Anti-ACV resistant strains activity of Fr.8.3

**Fig. 4:**
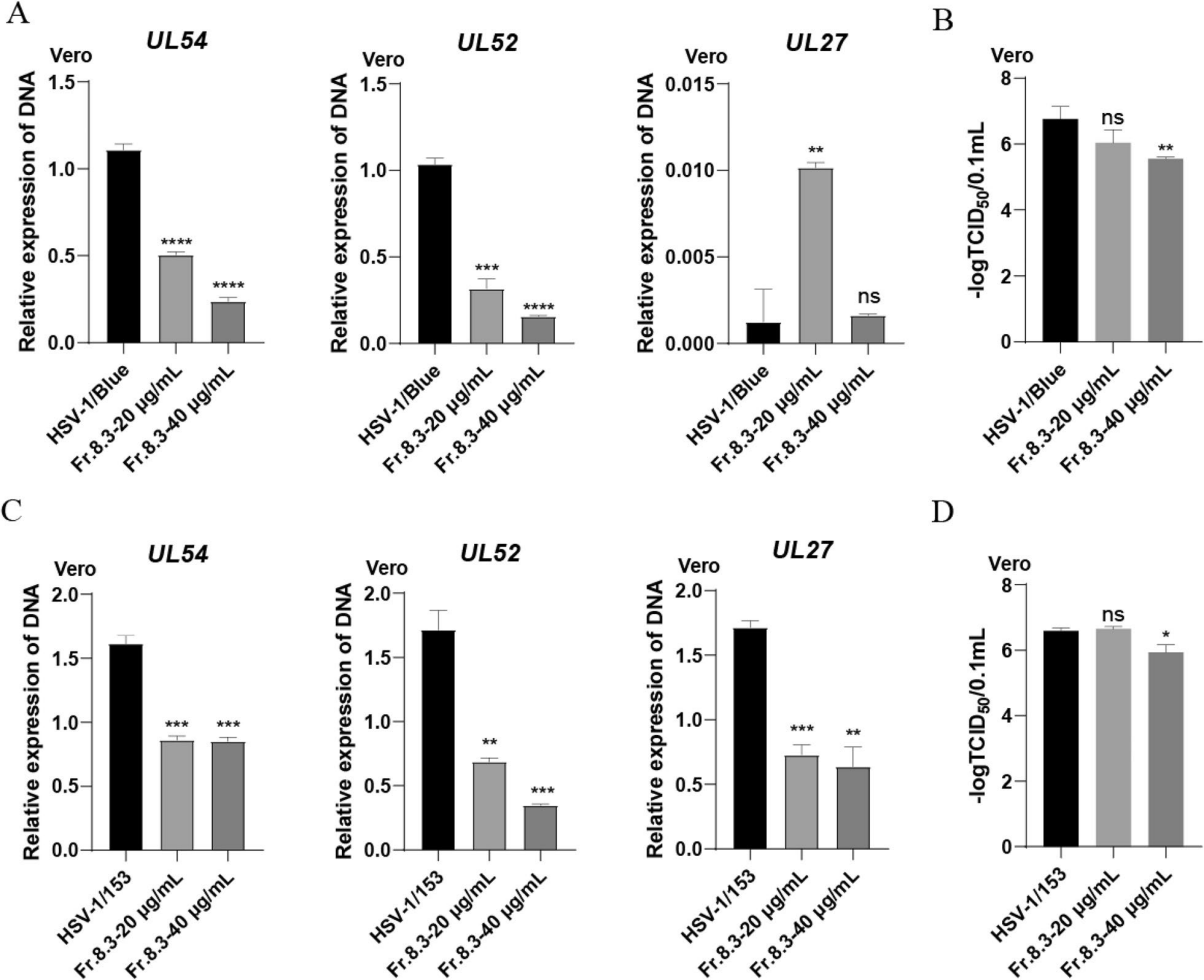
Anti-ACV resistant strains activity of Fr.8.3. **A** real-time fluorescence quantitative amplification reactions (Q-PCR) assessing the DNA level of the HSV-1 drug-resistant strain HSV-1/Blue, **B** viral titers of HSV-1/Blue, **C** real-time fluorescence quantitative amplification reactions (Q-PCR) assessing the DNA level of the HSV-1 drug-resistant strain HSV-1/153, **D** viral titers of HSV-1/153. The results presented were obtained from three independent experiments. Data represent the mean ± SD. Statistical differences were analyzed via Student’s t test, and a value of p ≤ 0.05 was considered significant (^*^p ≤ 0.05, ^**^p ≤ 0.01, ^***^p ≤ 0.001)

We then explored the activity of Fr.8.3 against ACV resistant strains. First, we detected the DNA expression levels of two HSV-1 drug-resistant strains, HSV-1/Blue and HSV-1/153, the results showed that Fr.8.3 significantly inhibited the genomic DNA copy number of both ACV-resistant strains at 40 μg/mL (Fig. 4A, C). In addition, we also detected titers for two ACV-resistant strains and showed that Fr.8.3 at 40 μg/mL was able to inhibit titers for both of them (Fig. 4B, D).

### Antiviral spectrum of Fr.8.3

**Fig. 5:**
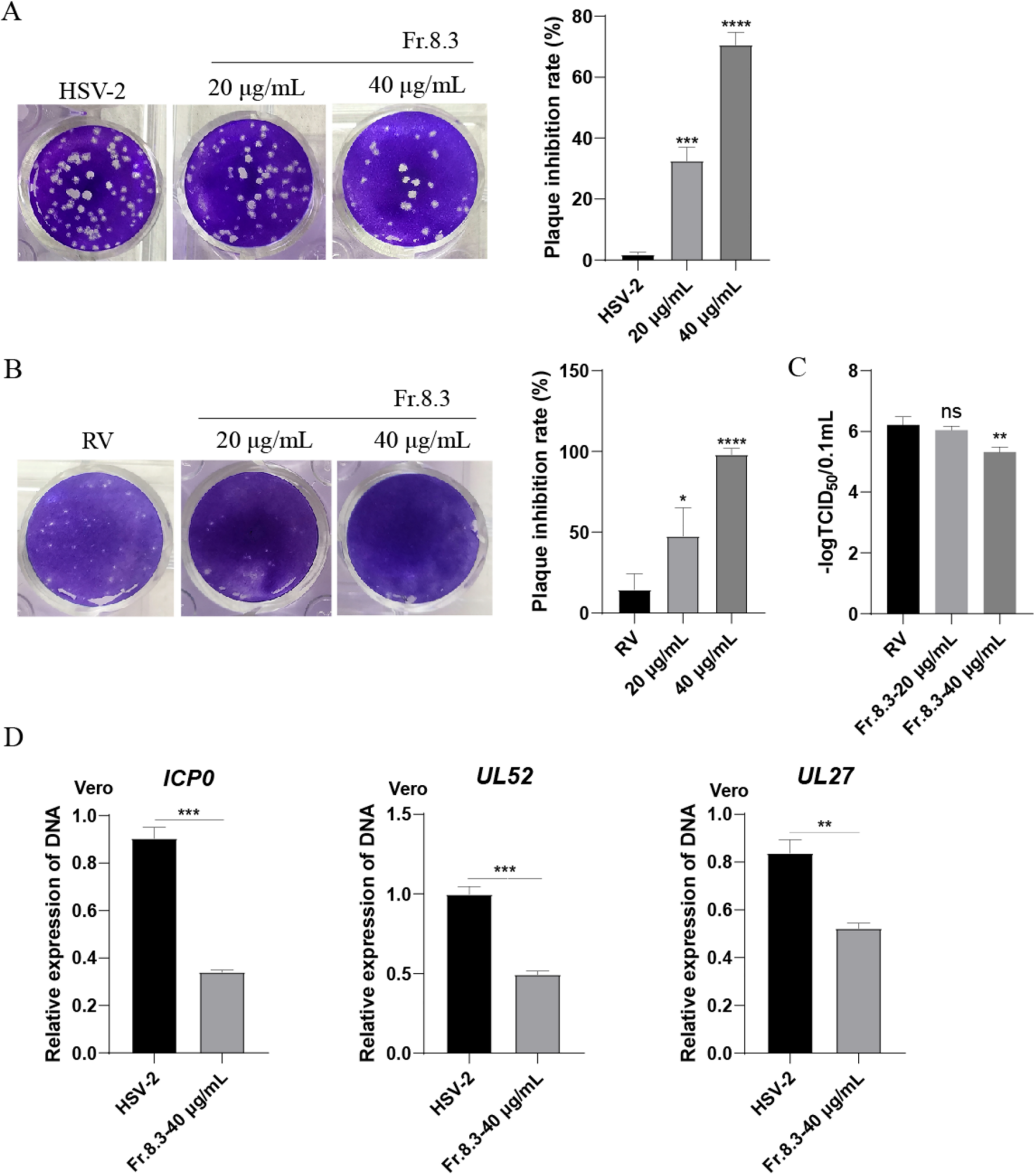
Antiviral spectrum of Fr.8.3. **A** Plaque reduction test of HSV-2, **B** plaque reduction test of RV, **C** viral titers of RV, **D** real-time fluorescence quantitative amplification reactions (Q-PCR) assessing the DNA level of HSV-2. The results presented were obtained from three independent experiments. Data represent the mean ± SD. Statistical differences were analyzed via Student’s t test, and a value of p ≤ 0.05 was considered significant (^*^p ≤ 0.05, ^**^p ≤ 0.01, ^***^p ≤ 0.001)

After observing the anti-HSV-1 activity of Fr.8.3, we wanted to clarify whether Fr.8.3 also has the same antiviral activity against other herpesvirus family members and other viruses, and therefore we conducted the following exploration. First, we performed viral plaque reduction test of HSV-2, the results showed that Fr.8.3 inhibited the number of plaques in HSV-2 at a concentration of 40 μg/mL, the inhibition rate of plaque was more than 70% (Fig. 5A). Then, we tested the antiviral activity of Fr.8.3 using rotavirus maintained in our laboratory, and the results of the plaque reduction test and viral titer determination consistently showed that Fr.8.3 had an anti-rotavirus effect. The rate of plaque inhibition was nearly 100%, and the RV titer was also significantly down-regulated (Fig. 5B, C). Furthermore, in order to more effectively describe the anti-HSV-2 activity of Fr.8.3, we detected the genomic DNA copy number of HSV-2, and the result showed that the DNA copy number of the three viruses decreased significantly after adding Fr.8.3 (Fig. 5D). In summary, Fr.8.3 shows an antiviral effect on a broad spectrum of viruses.

### Effects of Fr.8.3 on the direct inactivation, attachment and penetration of HSV-1

**Fig. 6:**
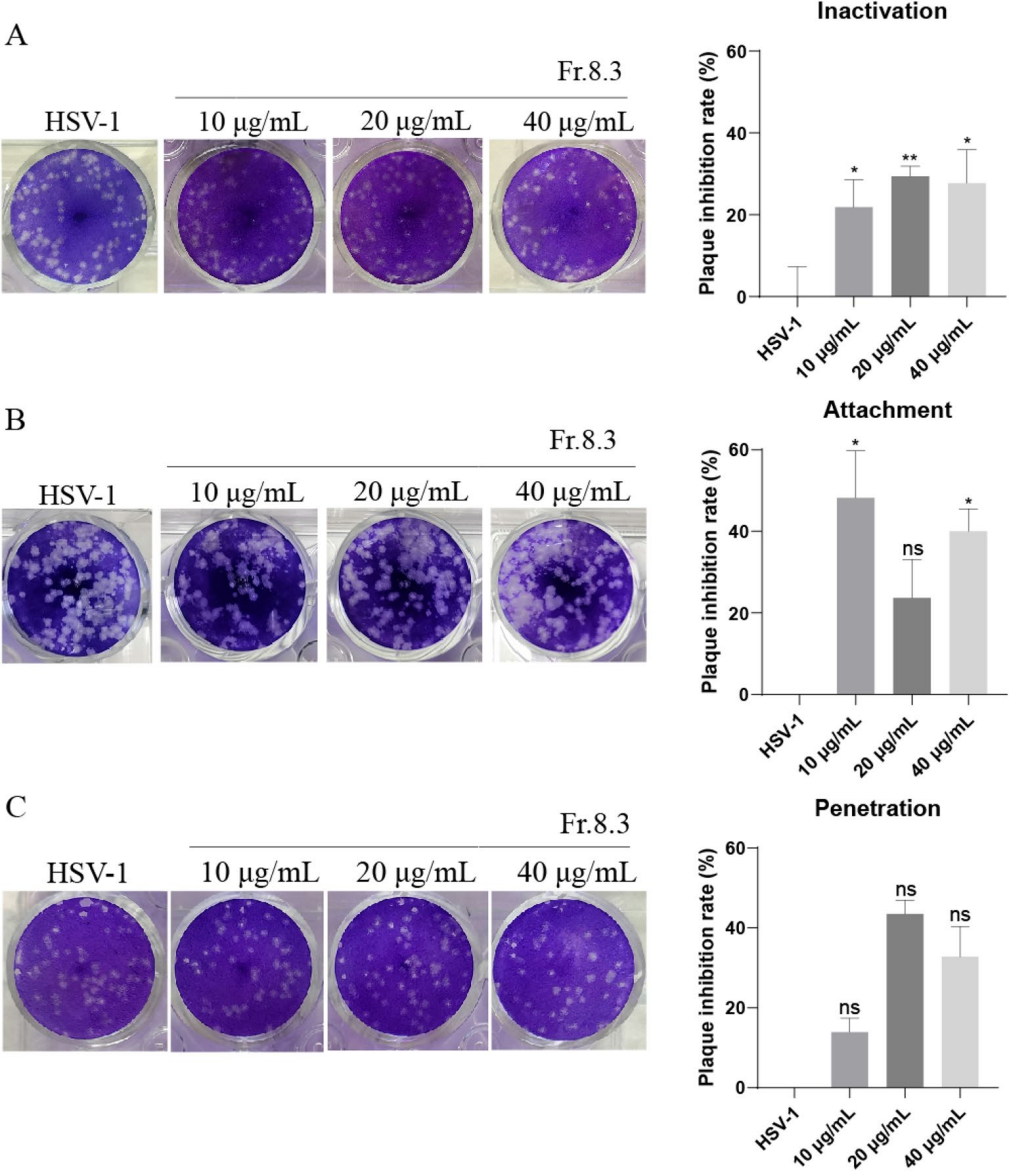
Direct inactivation, attachment and penetration of HSV-1 by Fr.8.3. **A** Direct inactivation of virus, **B** viral attachment, and **C** viral penetration assays. The plaque reduction test results presented were obtained from three independent experiments. Data represent the mean ± SD. Statistical differences were analyzed via Student’s t test, and a value of p ≤ 0.05 was considered significant (^*^p ≤ 0.05, ^**^p ≤ 0.01, ^***^p ≤ 0.001)

To further clarify the anti-HSV-1 mechanism of Fr.8.3, we carried out experiments to assess direct inactivation of virus, virus adsorption and virus penetration. The results demonstrated that Fr.8.3 at 40 μg/mL could not only directly inactivate HSV-1, but also inhibit the adsorption stages of HSV-1 (Fig. 6A, B). However, it showed no obvious inhibition of penetration stages of HSV-1 virus entry (Fig. 6C). These results indicate that Fr.8.3 exerts an anti-HSV-1 effect by directly acting on the virus or by inhibiting the viral adsorption stages, while its specific mechanism needs to be further explored.

### LC–MS analysis detected twelve compounds in the ethanolic extract of *Artemisia vulgaris L.*

**Fig. 7:**
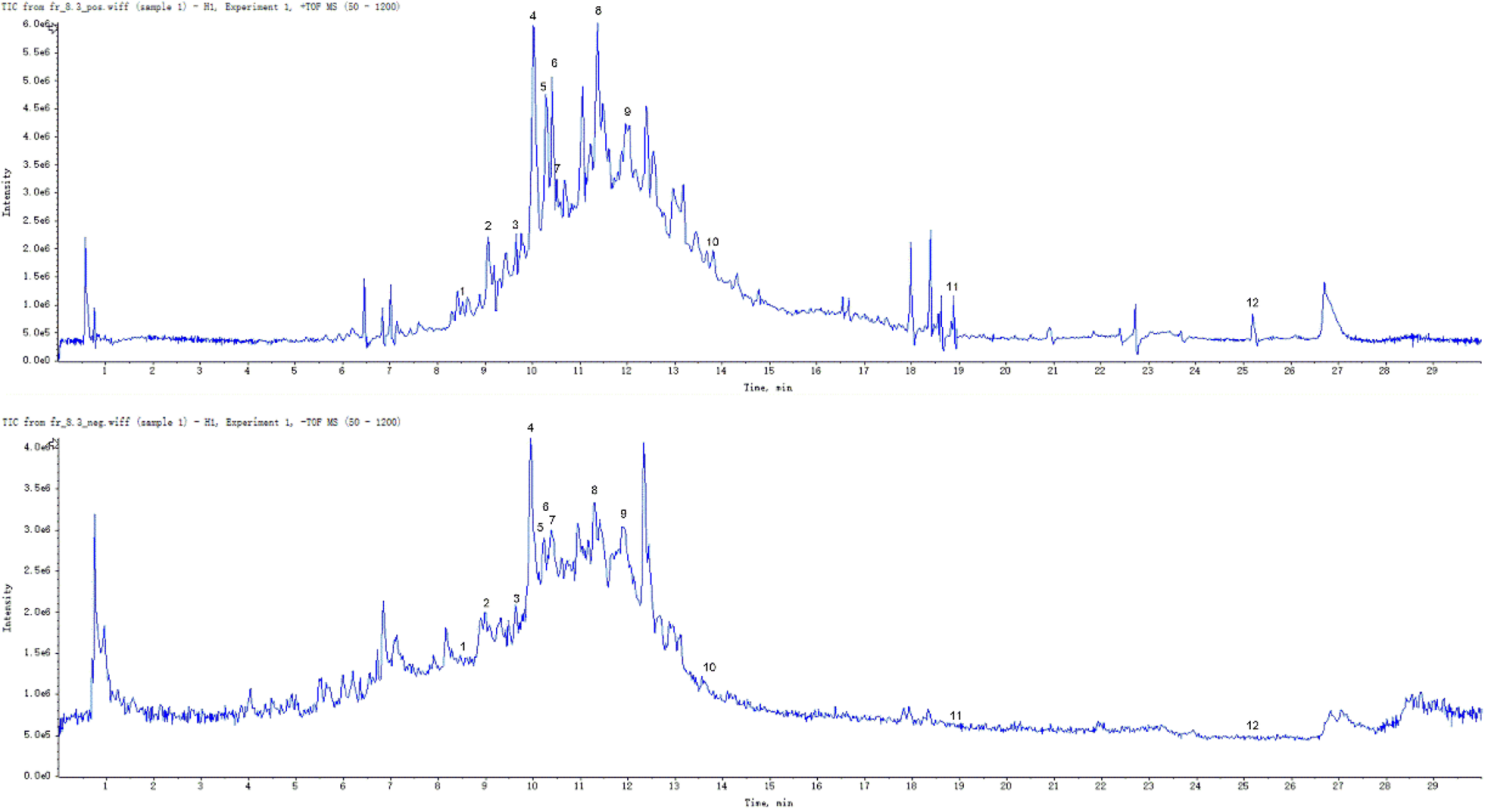
LC–MS analysis of the ethanolic extract of *Artemisia vulgaris L.*

In order to determine the main anti-HSV-1 components in the crude extract of Artemisia argyi, LC–MS analysis was performed on Fr.8.3. The results showed that several different molecules in the ethanolic extract from *Artemisia vulgaris L.* (Fig. 7). Among these, twelve compounds, including fenoprofen Ca, naloxone, austricine, OSTHOL, nabumetone and vinpocetine etc., were the major constituents in the *Artemisia vulgaris L.* leaf extract (Table 2).

**Table 2:** The major bioactive components detected and measured in the ethanolic extract of *Artemisia vulgaris L*. via LC–MS

Number	Retention time	Name	Molecular formula	Molecular weight	Area
1	8.526684	FenoprofenCa	C_15_H_14_O_3_	242.27	378760.219
2	9.068367	Naloxone	C_19_H_21_NO_4_	327.40	600392.750
3	9.662367	Austricine	C_15_H_20_O_5_	280.32	1339471.500
4	9.972366	OSTHOL	C_15_H_16_O_3_	244.28	850254.063
5	10.07572	9_10-alpha-Epoxy-eremanthin	C_15_H_18_O_3_	246.30	1781266.500
6	10.30738	Nabumetone	C_15_H_16_O_2_	228.29	1070968.000
7	10.43572	Deoxysappanone B 7,3"-dimethyl ether	C_20_H_20_O_6_	356.40	1207993.875
8	11.35207	Eriodictyol	C_15_H_12_O_6_	288.25	1393491.000
9	11.99175	Carboxyibuprofen	C_13_H_16_O_4_	236.26	806880.875
10	13.81513	Vinpocetine	C_22_H_26_N_2_O_2_	350.50	574186.188
11	18.8879	alpha-Lapachone	C_15_H_14_O_3_	242.27	792523.438
12	25.23938	Erucamide	C_22_H_43_NO	337.60	1626762.750

### Molecular docking results

Previous studies have shown that Fr.8.3 can directly inactivate HSV-1 and also inhibit the adsorption phase of HSV-1. Therefore, we wondered if Fr.8.3 inactivated the virus by inhibiting the HSV-1 adsorption process. Since the glycoproteins such as gB and gD on the surface of the virus capsule are closely related to the functions of virus adsorption, puncture and localization, we conducted target prediction through molecular docking in bioinformatics. To further confirm that one of the above components plays a major antiviral role, we performed molecular docking with the 12 components and target proteins gB, gD and their receptor molecules. Of course, further experiments will be required to explore and validate this hypothesis. We assume that the crude extract of single compounds also has an antiviral effect; therefore, the separation of crude extract for further extraction and purification is necessary, and this work is of great significance. The subsequent medicinal value and clinical significance of *Artemisia vulgaris L.* are very important and reflect the concept of the new use of an old medicine.

**Fig. 8:**
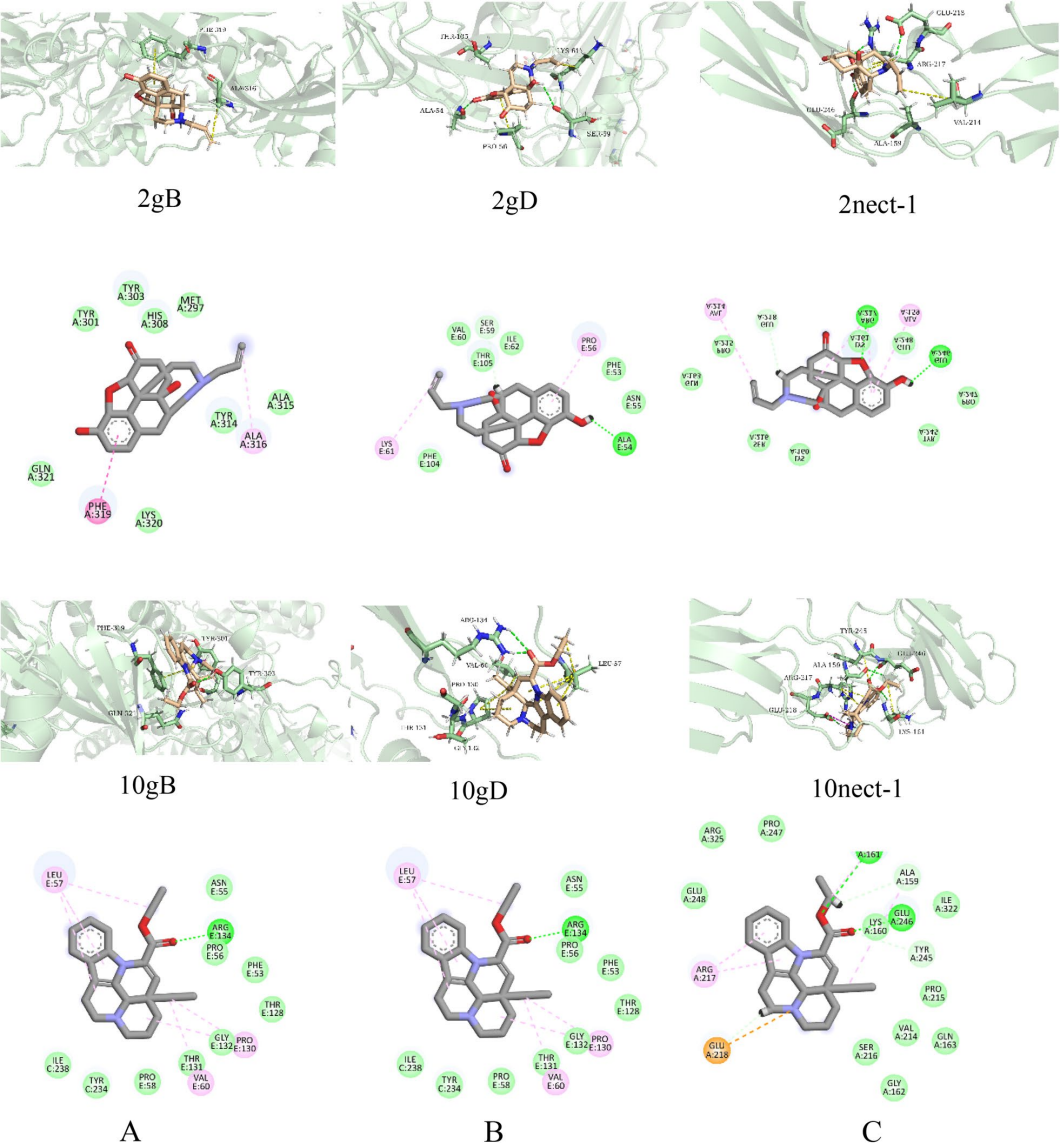
Two-dimensional interactions and binding poses of ligands 2 [naloxone] and 10 [vinpocetine] with **A** the extracellular domain of glycoprotein B of HSV-1 (gB, PDB code: 2GUM), **B** herpes simplex virus type 1 (HSV-1) glycoprotein D (gD, PDB code 3SKU), and **C** the crystal structure of nectin-1 (PDB code: 3U83)

First, we performed molecular docking for the 12 main components identified in the LC–MS analysis, and the docking score was the main parameter used to evaluate the interaction (Table 3). In other words, the lower the negative energy or fraction, the higher the free binding energy and the more favorable the thermodynamic interaction between the ligand and the protein. We predicted the different binding conformations of the receptor-binding ligands by the interactions of the above compounds with three target proteins. According to the obtained molecular docking results, Compounds 2 (naloxone) and 10 (vinpocetine) bound more stably to the 3 target proteins (gB and gD are HSV-1 proteins, and nect-1 is a host protein); that is, they were more compatible with receptor target proteins and could bind three at the same time. The specific data are shown in Table 3. In addition, the binding domain and two-dimensional interaction of the optimal ligands Compounds 2 and 10 are shown in detail in Fig. 8.

**Table 3:** Docking results of the top compounds with the lowest binding energies and docking score with three different receptors (energy values are in kcal/mol)

Ligand	gB			gD			nect-1		
	Docking score	Glide gscore	MMGBSA dG Bind	Docking score	Glide gscore	MMGBSA dG Bind	Docking score	Glide gscore	MMGBSA dG Bind
1	− 5.69	− 5.70	− 32.04	− 4.76	− 4.76	− 31.88	− 3.82	− 3.82	− 37.66
2	− 4.16	− 4.28	− 34.35	− 3.49	− 4.57	− 46.61	− 3.00	− 4.08	− 31.25
3	− 3.82	− 5.73	− 36.80	− 3.01	− 4.91	− 33.35	− 3.11	− 5.02	− 36.97
4	− 5.41	− 5.41	− 43.17	− 4.34	− 4.34	− 30.47	− 5.02	− 5.02	− 38.33
5	− 6.71	− 6.71	− 43.33	− 4.52	− 4.52	− 36.90	− 4.16	− 4.16	− 32.78
6	− 5.86	− 5.86	− 30.29	− 5.10	− 5.10	− 32.44	− 4.92	− 4.92	− 38.45
7	− 5.91	− 5.91	− 38.13	− 3.72	− 3.72	− 31.69	− 4.85	− 4.85	− 45.38
8	− 2.86	− 5.20	− 44.09	− 5.07	− 5.09	− 32.63	− 3.25	− 5.93	− 36.07
9	− 5.17	− 5.17	− 33.79	–	–	–	− 5.28	− 5.28	− 30.64
10	− 4.79	− 5.33	− 42.12	− 3.65	− 4.20	− 37.62	− 4.14	− 4.44	− 35.95
11	− 6.00	− 6.00	13.01	− 4.77	− 4.77	− 31.51	− 4.93	− 4.93	− 30.56
12	–	–	–	− 1.67	− 1.67	− 31.26	− 2.74	− 2.74	− 49.85

## Discussion

Herpes simplex virus type I (HSV-1) is a double-stranded DNA enveloped virus that mainly infects the mouth, skin and eyelids, causing gingival stomatitis, dermal herpes and keratoconjunctivitis. HSV-1 also causes herpes simplex encephalitis (HSE), which has a mortality rate of up to 70% [37]. Even more worrisome is the fact that herpes simplex viruses remain latent in the host's peripheral nervous system for life and can never be eradicated. Since the discussion of herpes simplex virus encephalitis (HSE) by the Mathewson Commission in 1926 and the subsequent description of histopathological changes, herpes simplex virus infection has become one of the most common causes of sporadic fatal encephalitis [38]]. HSE underlies the viral origin of diseases of the Central Nervous System (CNS). Despite being able to be treated by the use of antiviral approaches, HSE remains a common CNS infectious disease [39]. Since most patients have serious neuropsychological sequelae after taking the clinical drug acyclovir and drug resistance is frequent, it is necessary to develop specific and efficient clinical drugs, in particular from natural compounds for anti-HSV-1 therapy.

The bioactive ingredients of *Artemisia vulgaris L.* have many different pharmacological effects, and their application value is very wide. However, there are few reports on the antiviral effects of *Artemisia vulgaris L.*, especially anti-herpesvirus effects. Our results showed that the extracts Fr.8.3 had a significant inhibitory effect on viral replication. Also, the results of molecular docking suggest that the extracts may have an inhibitory effect on virus adsorption and entry into cells. At present, the main problem we are facing is how to continue to separate and extract single pure monomer compounds from the crude extract of *A. vulgaris* and further explore the mechanism of the monomer compounds, which will be an innovative work to screen out the components of *A. vulgaris* with good anti-herpesvirus activity through the method of antiviral drug screening, which is of great significance. This study has not only enhanced the potential application value of Chinese herb *A. vulgaris* but also laid a theoretical foundation for the development of new clinical drugs against herpesviruses and provided new ideas.

## Data Availability

The research data generated from this study are included in the article and additional files.

## Abbreviations

HSV-1: Herpes simplex virus type 1
HSV-2: Herpes simplex virus type 2
HSE: Herpes simplex virus encephalitis
*A. vulgaris L.*: *Artemisia vulgaris L.*
HIV: Human immunodeficiency virus
HBV: Hepatitis B virus
ACV: Acyclovir
VCV: Valaciclovir
FCV: Famiclovir
AAEO: Essential oil of *Artemisiae argyi*
AAFP: Polysaccharide of *Folium Artemisiae argyi*
FEAA: Flavone of *Artemisia argyi*
MeOH: Methyl alcohol
Vero: African green monkey kidney cells
DMEM: Dulbecco’s modified Eagle’s medium
FBS: Fetal bovine serum
CPE: Cytopathic effect
MOI: Multiplicity of infection
PFU: Plaque-forming unit
PBS: Phosphate buffered saline
GAPDH: Glyceraldehyde-phosphate dehydrogenase
Q-PCR: Quantitative real-time—polymerase chain reaction
